# Quantification of amylose, amylopectin, and β-glucan in search for genes controlling the three major quality traits in barley by genome-wide association studies

**DOI:** 10.3389/fpls.2014.00197

**Published:** 2014-05-15

**Authors:** Xiaoli Shu, Søren K. Rasmussen

**Affiliations:** ^1^Key Laboratory of the Ministry of Agriculture for Nuclear Agricultural Sciences, Institute of Nuclear Agricultural Sciences, Zhejiang UniversityHangzhou, China; ^2^Department of Plant and Environmental Sciences, Faculty of Sciences, University of CopenhagenFrederiksberg, Denmark

**Keywords:** *Hordeum vulgare*, GWAS, β-glucan, amylose, amylopectin, co-regulation

## Abstract

Genome-wide association studies (GWAS) for amylose, amylopectin and β-glucan concentration in a collection of 254 European spring barley varieties allowed to identify 20, 17, and 21 single nucleotide polymorphic (SNP) markers, respectively, associated with these important grain quality traits. Negative correlations between the content of amylose and β-glucan (*R* = −0.62, *P* < 0.01) and amylopectin and β-glucan (*R* = −0.487, *P* < 0.01) were found in this large collection of spring barley varieties. Besides *HvCslF6, amo1 and AGPL2, sex6*, and *waxy* were identified among the major genes responsible for β-glucan, amylose and amylopectin content, respectively. Several minor genes like *HvGSL4, HvGSL3*, and *HvCesA6*, PWD were also detected by GWAS for the first time. Furthermore, the gene encoding β-fructofuranosidase, located on the short arm of chromosome 7H at 1.49 cM, and SRF6, encoding “leucine-rich repeat receptor kinase protein” on chromosome 2 H, are proposed to be new candidate genes for amylopectin formation in barley endosperm. Several of the associated SNPs on chromosome 1, 5, 6, and 7H mapped to overlapping regions containing QTLs and genes controlling the three grain constituents. In particular chromosomes 5 and 7H carry many QTLs controlling barley grain quality. Amylose, amylopectin and β-glucan were interacted among each other through a metabolic network connected by UDP showing pleiotropic effects. Taken together, these results showed that cereal quality traits related each other and regulated through an interaction network, the identified major genes and genetic regions for amylose, amylopectin and β-glucan is a helpful for further research on carbohydrates and barley breeding.

## Introduction

Over the last 30 years, glycemic index (GI) of food has been taken as guideline for controlling diabetes, obesity and other nutritional related diseases (Kirpitch and Maryniuk, 2011) and due to its low GI, barley is being promoted for healthy food products. Barley is an excellent source of complex carbohydrates and β-glucans and ranks the fourth most widely cultivated and utilized cereal worldwide after maize, rice and wheat (Newton et al., 2011). The main carbohydrate in barley is starch ranging from 62 to 77% of the grain dry weight. Amylose and amylopectin are the two components of starch, according to amylose content, barley can be classified into normal type (25–27% amylose), waxy type (non-detectable and below 5% amylose), and high-amylose type (>35% amylose). Variations in the amylose content can affect the physicochemical and functional properties of starch which may in turn affect its utilization in various industrial applications. High-amylose barley contributes toward a low GI and also promotes bowel health (Asare et al., 2011) and hence it is an excellent source of dietary fiber. Barley waxy type starches which are almost devoid in amylose were shown to have excellent freeze/thaw stability making them suitable in frozen foods (Bahatty and Rossangel, 1997).

β-glucan is the primary component of the cell wall in the starchy endosperm of barley accounting for around 75% of endosperm cell wall mass. β-glucan consists of D-glucose monomers linked by different β-glycosidic linkages such as 1,3/1,4 bonds and ranges from 4 to 10% of the dry weight across barley varieties (Brennan and Cleary, 2005; Burton and Fincher, 2012). Although the levels of β-glucans contribute little to the total grain weight, they have great influence on the nutritional value, functionality and uses of barley. β-glucans are regarded to be the most important factor determining malting potential and brewing yield by regulating the rate of endosperm modification and the viscosity of wort during brewing (Brennan and Cleary, 2005). High β-glucan and waxy phenotypes are regarded unfavorable for brewing and feed stuff, but they are considered to be a valuable source of soluble fiber in human diets showing potential functions in reducing GI and serum cholesterol levels, flattening the postprandial blood glucose levels and insulin rises (Thondre and Henry, 2009; Ahmad et al., 2012). The ability of β-glucan to lower the GI is dependent on the barley starch type (Finocchiaro et al., 2012) and other grain chemicals, especially the total content of fiber (Aldughpassi et al., 2012).

The amylose and β-glucan content are primarily under genetic control but environmental factors especially during grain filling has a smaller contribution to the final grain composition (Baik and Ullrich, 2008). Amylose concentrations in barley are controlled mainly by the *amo1* (amylose) and *waxy* loci mapped on chromosome 1HS (5S) and chromosome 7HS (1S), respectively; another locus *sex6* on 7H is also responsible for amylose content (Morell et al., 2003). Several quantitative trait loci (QTLs) controlling amylose accumulation had been further mapped on chromosome 1, 5, and 7H, respectively (Islamovic et al., 2012).

The amount of β-glucan was shown to be controlled by several QTLs located on chromosome 2 and 5H (Han et al., 1995), 4H (Gao et al., 2004; Wei et al., 2009), and 7H (Li et al., 2008). Cellulose synthase (*CesA*, EC2.4.1.12), cellulose synthase-like (*Csl*) superfamily and glucan synthase-like (*Gsl*) (EC2.4.1.34) gene family are involved in the synthesis of most β-linked polysaccharides of the cell wall (Farrokhi et al., 2006). *Csls* are believed to encode enzymes that synthesize the backbone of various non-cellulosic β-linked polysaccharides and have been classified into 8 gene families, designated *CslA* to *CslH* (Farrokhi et al., 2006; Dhugga, 2012). A cluster of *CslF* genes has been found on chromosome 2H (Burton et al., 2006) and valuable single nucleotide polymorphism (SNP) markers of the *HvCslF6* gene on chromosome 7H have been developed (Cory et al., 2012). Several QTLs for β-glucan content overlap with QTLs for amylose accumulation (Islamovic et al., 2012) probably/likely due to co-localization or interaction between genes.

Here we use genome association studies to map loci and genetic regions responsible for amylose, amylopectin and β-glucan content, respectively, using a large spring barley variety collection already genotyped with SNPs. To our knowledge, there are no reports on simultaneous genetic analysis of β-glucan, amylose and amylopectin content by genome-wide association studies (GWAS). This approach can provide new insights into the regulation and interaction mechanisms among genes involved in carbohydrate metabolism in the starchy barley endosperm, and thus highlight markers for crop improvement and breeding of barley varieties with suitable amylose and β-glucan content. As rice genome sequencing have been completed and most genes have been annotated (http://rice.plantbiology.msu.edu/), by analyzing each candidate gene according to syntenous and comparative genomics with rice we were able to elucidate part of the co-regulation network among the biosynthetic pathways of amylose, amylopectin and β-glucan at the same time.

## Materials and methods

### Germplasm and genotyping

A population of 254 spring barley varieties obtained from the *Hordeum vulgare* Core Collection of EXBARDIV (http://www.erapg.org/publicitem.m?key=everyone&pgid=18622&trail=/everyone/9587/18624/18614/18622) (Tondelli et al., 2013) was used for amylose and β-glucan analysis. All material was grown at Scottish Crop Research Institute, now James Hutton Institute Dundee, Scotland (UK) 2010. After harvesting, grains were air-dried and grounded into flour, passed through 65 mesh sieves and stored at 4°C in sealed plastic bags until use. All varieties had been genotyped by TraitGenetics, GmbH, Gatersleben (Germany) using the iSelect set of 7864 SNP markers (Comadran et al., 2012).

### Amylose, amylopectin, and β-glucan quantification

Amylose and amylopectin content was measured according to the Chinese National standard method GB7648-87 and Wang et al. (2010), respectively, with minor modifications: 10 ± 0.1 mg barley grain flour were weighed into a 14 mL tube with caps, dispensed in 0.1 mL 95% ethanol, and treated with 0.9 mL 1 M NaOH for 16 h at room temperature. The samples were mixed and 10 μL supernatant were pipetted into 96-well plates, 190 μL freshly prepared I_2_-KI solution (3% iodine solution diluted 100 times in 0.01 M HCl before use) were added and incubated for 10 min, potato amylose and amylopectin (Sigma-Aldrich Co.) were treated in the same way and used to construct the standard curve. Amylose and amylopectin content was measured according to the absorbance at 620 nm (OD_620_) and OD_550_–OD_740_, respectively. The results were calculated based on sample weight. β-glucan content was quantified according to the assay for barley using Megazyme mixed-linkage β-glucan kit (K-BGLU, Megazyme). All analyses were run in triplicate with independent samples.

### Association analysis

Association analyses were all conducted as described in a recent study of diversity and history of European barley (Tondelli et al., 2013). The genetic structure of 254 spring barley cultivars was analyzed using Structure 2.3.3 (Pritchard et al., 2000) with 6810 SNPs. Linkage disequilibrium analyses of the 6810 SNPs were conducted with TASSEL 3.0.9 (http://www.maizegenetics.net/tassel). We employed a naïve model without correction for population structure, a Q model and a Q+K model, both to correct for population structure. For the Q+K model we employed the Kinship matrix and 4 principal components. We also examined P+K models based on Bayesian information criterion (BIC) (Zhu and Yu, 2009). Markers were declared significant at the *P* = 0.001 [−Log (*P*) = 3] threshold for amylose and amylopectin and *P* = 0.002 (−Log (*P*) = 2.75] for β-glucan with the selected model according to a liberal approach proposed by Chan et al. (2010).

### Synteny to rice and *Brachypodium* genomes

The homologous gene of barley SNP(s) defining the QTL was located in the genome zipper (Mayer et al., 2011) and Strudel (Milne et al., 2010). SNPs with significant associations were used to search for genes functionally related to starch and glucan metabolism according to the annotations of barley genes (http://barleyflc.dna.affrc.go.jp) and also by taking advantage of the synteny between barley and the annotated rice and *Brachypodium* genomes, respectively. These SNPs or genes close to the SNPs in barley were considered to be candidate genes. Probabilistic functional gene networks of candidate genes in starch metabolism were integrated based on the data from KEGG (http://www.kegg.com) and RiceCyc (http://pathway.gramene.org).

## Results

### Trait variations

**Figure 1:**
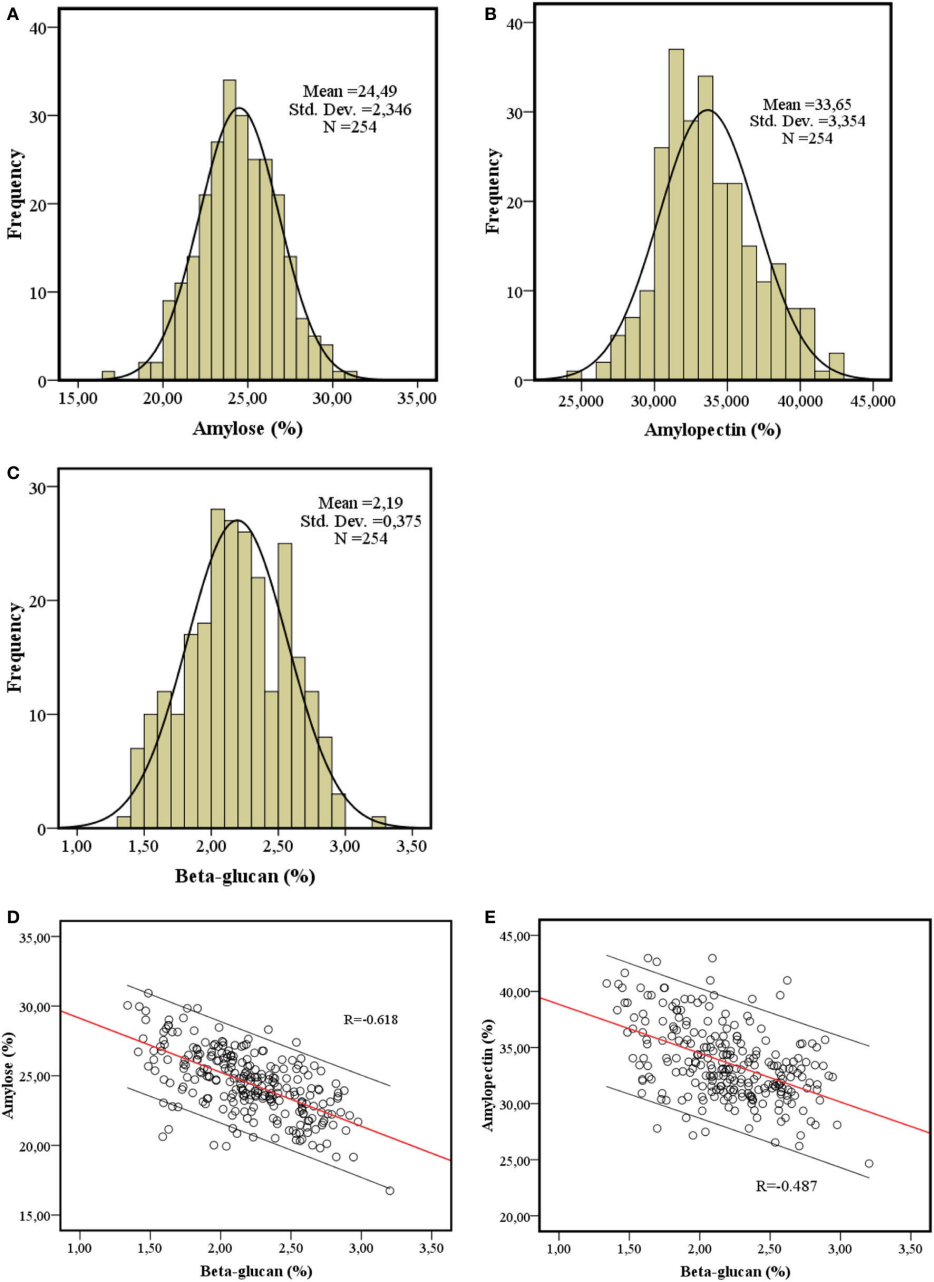
**Quantification of barley grain constituents**. The distribution of amylose content **(A)**, amylopectin content **(B)** and β-glucan content **(C)** and the correlation between amylose content and β-glucan content **(D)**, amylopectin content and β-glucan content **(E)** in 254 barley spring cultivars.

The quantification of amylose, amylopectin and β-glucan in the spring barley collection showed normal distributions (Figure 1) and varied from 16.74 to 30.92, 26.47 to 42.97, and 1.34 to 3.20%, respectively. The average amylose content in 212 2-row and 42 6-row types was 24.54 ± 2.36 and 24.35 ± 2.31%, respectively, and the average β-glucan content in 2-row and 6-row types was 2.20 ± 0.38 and 2.15 ±0.35%, respectively (Table S1). Data were consistent with the variances in the whole panel and showed no correlation with geographic origin, year of release or row-type (data not shown). The amylopectin content had the same distribution trend with average content of 33.79 ± 3.42 and 32.92 ± 2.91% in 2-row and 6-row types, respectively. The analysis of the 254 spring barley varieties showed that β-glucan content is significantly negatively correlated with both amylose content (*R* = −0.62, *P* < 0.01) and amylopectin (*R* = −0.487, *P* < 0.01) (Figures 1D,E).

### Marker-trait association and model comparison for association analysis

**Figure 2:**
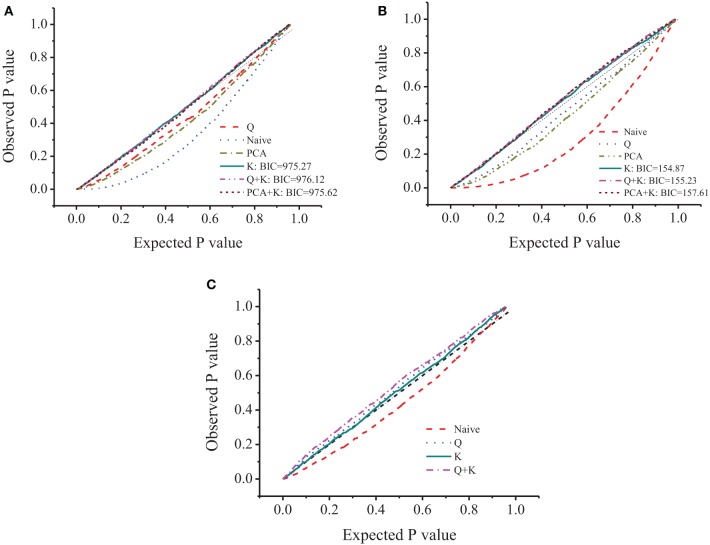
**P-P plot of different model for amylose (A), β-glucan (B), and amylopectin (C)**.

Tondelli et al. (2013) evaluated this barley collection for population structure and linkage disequilibrium, and arrived at *K* = 4 as the number of principal components in the Q+K model. In order to decrease the false negative associations and still maintain a strong prediction power, the optimization of the model used for the specific case under evaluation was necessary. Naïve, Q, PCA, Kinship (K), Q+Kinship (Q+K), and PCA+Kinship (PCA+K) were performed and interpreted based on BIC and the fitting plot (observed against expected probability, PP-plot). Among the 6 models, naïve, Q and PCA showed skewed P−P plot, the other 3 models equipped with Kinship showed better fitting plot and similar BIC value. Because K model showed the smallest BIC with better fitting plot, the association analyses were based on K model (Figure 2) for all three traits.

### Mapping results and allelic effects

**Figure 3:**
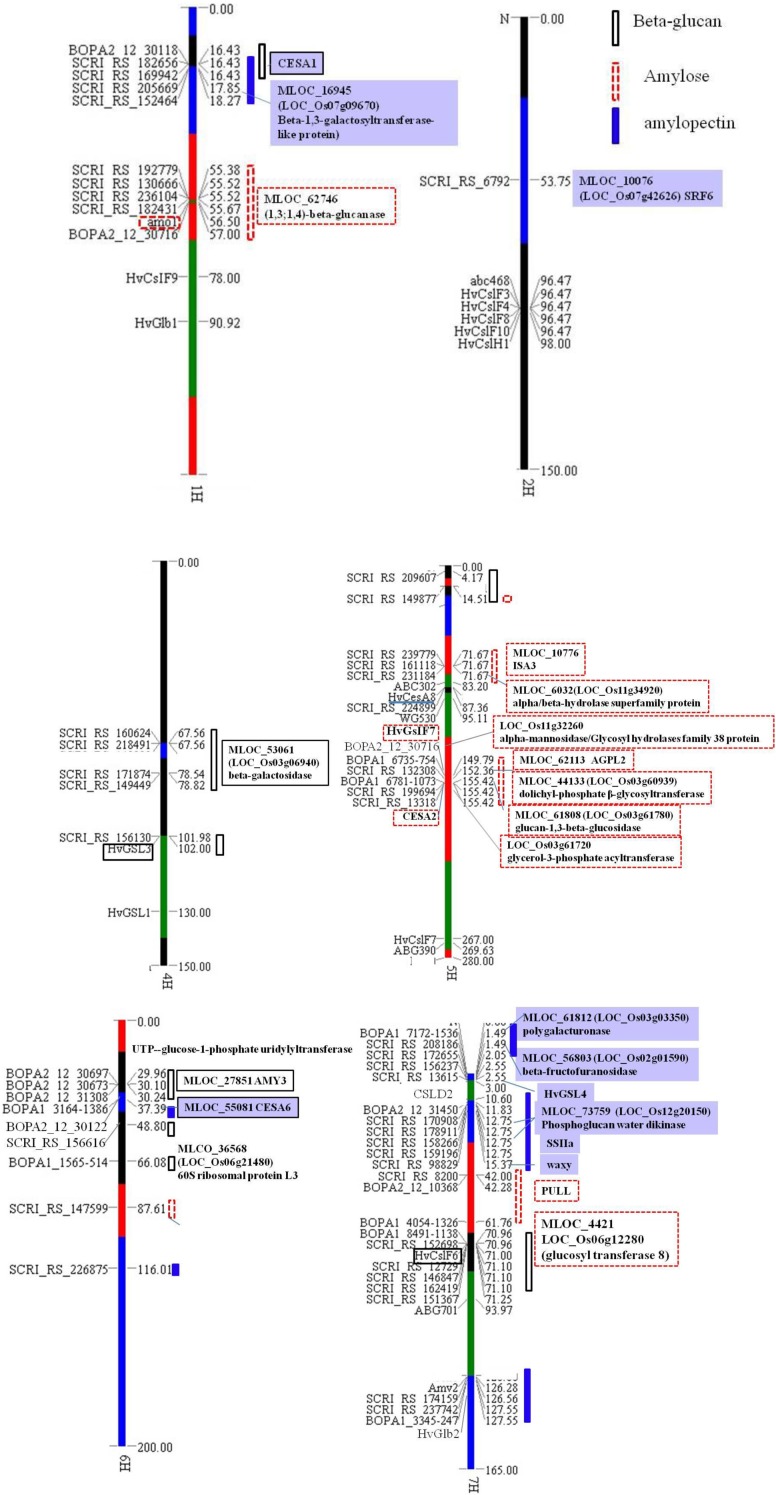
**Major SNPs for amylose, amylopectin and β-glucan and their orthologous genes or close genes**. SNPs regions responsible for β-glucan are indicated by a solid box, for amylose are indicated by a dashed box, and for amylopectin are indicated by a bar. The candidate genes were boxed with solid lines, dashed lines and solid frames for β-glucan, amylose and amylopectin, respectively. The maps were draw by software GTT2 and all genetic positions are comparable to the map of OPA 2009 concensus (www.gramene.org/db/cmap/viewer?changeMenu=1).

Significant marker-trait associations for β-glucan content using a threshold of −Log (*P*) > 2.7 (*P* < 0.002) was found for 21 SNPs, and 20 and 17 significant SNPs were identified for amylose and amylopectin, respectively, using a threshold of −Log (*P*) > 3 (*P* < 0.001) level (Table 1). Most of the SNPs identified accounted for 5–7% of the variation, except 2 SNPs for amylopectin, SCRI_RS_6792 and SCRI_RS_208186 that accounted for 14.5 and 46.9% of the variation, respectively. Almost 40% of the SNPs were located on chromosome 7H, the remaining are distributed on 1, 4, 5, and 6H. The SNP for amylopectin accounting for 14.5% of the variation was the only located on chromosome 2H (Table 1). Using the genetic position in Table 1, all SNPs were included in the OPA 2009 consensus map (Figure 3) together with previously mapped genes relevant to this study in order to identify overlap among the three traits and known genes and QTLs.

**Table 1:** **Associated SNPs for β-glucan, amylose and amylopectin content with the highest possibility called by K model**.

**Traits**	**SNPs**	**CH**	**Position**	***P*-value**	***R*^**2**^**	**Alleles**	**Annotation**
β-glucan	BOPA2_12_30118	1H	16.43	1.2E-03	0.050	C/G	Ankyrin repeat domain containing protein 28
	SCRI_RS_169942	1H	16.43	7.01E-04	0.054	G/A	–
	SCRI_RS_160624	4H	67.56	1.21E-03	0.050	T/C	Methyladenine glycosylase domain containing protein
	SCRI_RS_218491	4H	67.56	1.82E-03	0.046	C/T	Heat shock protein DnaJ
	SCRI_RS_171874	4H	78.54	1.12E-03	0.050	C/T	–
	SCRI_RS_149449	4H	78.82	6.19E-04	0.055	C/T	Protein of unknown function DUF620 family protein
	SCRI_RS_156130	4H	101.98	1.67E-03	0.047	A/C	1,3-beta-glucan synthase
	SCRI_RS_209607	5H	4.17	1.7E-03	0.050	G/A	–
	SCRI_RS_149877	5H	14.51	1.42E-03	0.051	T/C	Terpene synthase
	SCRI_RS_224899	5H	87.36	1.4E-03	0.048	A/G	Expressed protein
	BOPA2_12_30697	6H	29.96	8.79E-04	0.052	A/C	RNA recognidtion motif ocntaining protein
	BOPA2_12_30673	6H	30.10	8.79E-04	0.052	A/G	Division protein
	BOPA2_12_31308	6H	30.24	8.79E-04	0.052	G/C	Pentatricopeptide
	SCRI_RS_156616	6H	48.80	1.74E-03	0.047	A/G	Expressed protien
	BOPA1_1565-514	6H	66.08	1.92E-03	0.046	G/A	60s ribosomal protein L31
	BOPA1_8491-1138	7H	70.96	4.52E-04	0.058	A/G	MBTB15-Bric-a-Brac, Tramtrack
	SCRI_RS_152698	7H	70.96	4.52E-04	0.058	C/T	Kelch related domain containing protein
	SCRI_RS_12729	7H	71.10	6.13E-04	0.055	T/C	Protein of unknown function DUF23 family protein
	SCRI_RS_146847	7H	71.10	6.13E-04	0.055	C/T	Hypothetical conserved gene
	SCRI_RS_162419	7H	71.10	6.13E-04	0.055	A/G	Similar to Topoisomerase-like protein
	SCRI_RS_151367	7H	71.25	5.15E-04	0.057	G/A	AT hook motif domain containing protein
Amylose	SCRI_RS_192779	1H	55.38	3.34E-04	0.058	A/G	Copine, putative
	SCRI_RS_130666	1H	55.52	3.34E-04	0.058	C/A	Conserved hypothetical protein
	SCRI_RS_236104	1H	55.52	3.34E-04	0.058	G/A	Expressed protein
	SCRI_RS_182431	1H	55.67	3.34E-04	0.058	G/A	Conserved hypothetical protein
	SCRI_RS_149877	5H	14.51	3.59E-04	0.061	T/C	Terpene synthase
	SCRI_RS_161118	5H	71.67	2.47E-04	0.061	T/G	Leucine-rich repeat family protein
	SCRI_RS_231184	5H	71.67	2.47E-04	0.061	A/C	Stripe rust resistance protein Yr10, putative, expressed
	SCRI_RS_239779	5H	71.67	2.47E-04	0.061	C/T	–
	BOPA1_6735-754	5H	149.79	3.92E-04	0.059	G/C	Expressed protein
	SCRI_RS_132308	5H	152.36	2.20E-04	0.067	A/G	Dolichyl-phosphate beta-glycosyltransferase
	BOPA1_6781-1073	5H	155.42	3.04E-04	0.059	A/G	OsSPL6-SBP-bpx gene family member
	SCRI_RS_13318	5H	155.42	4.08E-04	0.057	T/C	Expressed protein
	SCRI_RS_199694	5H	155.42	2.88E-04	0.059	A/G	Expressed protein
	SCRI_RS_147599	6H	87.61	6.45E-04	0.053	T/C	RPA1A-Putative single-stranded DNA binding complex subunit 1
	SCRI_RS_8200	7H	42.00	7.57E-04	0.051	A/G	LTP family protein
	BOPA2_12_10368	7H	42.28	7.57E-04	0.051	C/A	RNA-binding protein-like
	BOPA1_4054-1326	7H	61.76	4.62E-04	0.056	G/A	Aquaporin protein
	BOPA2_12_30716	5H	NA	7.24E-04	0.052	T/A	Lysosomal alpha-mannosidase
	SCRI_RS_108855	NA	NA	4.08E-04	0.057	G/A	CGMC_GSK.1–CGMC includes CDA, MAPK, GSK3, and CLKC kinases/glycogen synthase kinase 3-related GSK3
	SCRI_RS_182201	NA	NA	4.08E-04	0.057	G/T	NB-ARC domain containing protein
Amylopectin	BOPA2_12_30122	6H	NA	2.41E-04	0.061	G/A	Similar to JHL07K02.7 protein
	SCRI_RS_182656	1H	16.43	4.67E-04	0.056	G/A	–
	SCRI_RS_205669	1H	17.85	4.69E-04	0.056	C/T	Galactosyltransferase family protein
	SCRI_RS_6792	2H	53.75	5.77E-09	0.148	C/T	Strubbelig receptor family 6/Leucine-rich repeat receptor kinase-like protein SRF6
	BOPA1_3164-1386	6H	37.39	9.62E-04	0.050	G/A	Cellulose synthase CesA1
	SCRI_RS_208186	7H	1.49	1.15E-30	0.469	G/A	Glycosyl hydrolases
	BOPA1_7172-1536	7H	1.49	6.89E-04	0.055	C/G	Glycoside hydrolase, family 28, putative, expressed
	SCRI_RS_172655	7H	2.05	5.57E-05	0.073	A/C	–
	SCRI_RS_13615	7H	2.55	4.31E-04	0.056	A/G	B3 DNA binding domain containing protein
	SCRI_RS_158266	7H	12.75	6.64E-05	0.071	C/T	Phosphoglucan water kinase
	SCRI_RS_159196	7H	12.75	6.64E-05	0.071	T/C	Expressed protein
	SCRI_RS_170908	7H	12.75	7.01E-04	0.052	C/T	Phosphoglucan water kinase
	SCRI_RS_178911	7H	12.75	7.01E-04	0.052	C/T	WD domain, G-beta repeat domain containing protein
	SCRI_RS_98829	7H	15.37	8.81E-04	0.050	T/C	Conserved hypothetical protein
	SCRI_RS_174159	7H	126.56	7.93E-04	0.053	A/C	Similar to Aldehyde oxidase-2
	SCRI_RS_237742	7H	127.55	8.75E-04	0.050	C/T	Expressed protein
	BOPA1_3345-247	7H	127.55	9.59E-04	0.050	G/A	Homeobox protein knotted-1

Three significant SNP regions associated with amylopectin were detected on chromosome 7H. Five SNPs detected at the region from 12.75 to 15.37 cM on 7H are very close to the *waxy* locus. Besides this locus, new SNPs have been identified for candidate genes controlling amylopectin content in the present GWAS. The SNP at 1.49 cM (SCRI_RS208186) was detected for the first time and accounted for about 46.9% of the variation (Table 1), which should represent the major locus responsible for amylopectin content. The significant SNP region from 126.56 to 127.55 cM on chormose 7H might be linked to *Amy2* locus, which had been mapped at 126 cM.

Barley chromosome 7H also has 6 SNPs significant for β-glucan content located around 71 cM showing the highest genetic effect and each SNP accounted for 5–6 % variations (Table 1, Figure 3). These SNPs map close to cellulose synthase like gene *HvCslF6* which had been mapped around 71 cM according to the comparative barley map (http://wheat.pw.usda.gov).

Barley chromosome 1H have four possible SNPs for amylose content located close to the *amo1* gene located at 56.5 cM between marker *lca1* and *Js074* (Barley BinMap 2005, http://wheat.pw.usda.gov) close to 60 cM on the map of OPA2009 consensus.

### Candidate genes for β-glucan content

**Figure 4:**
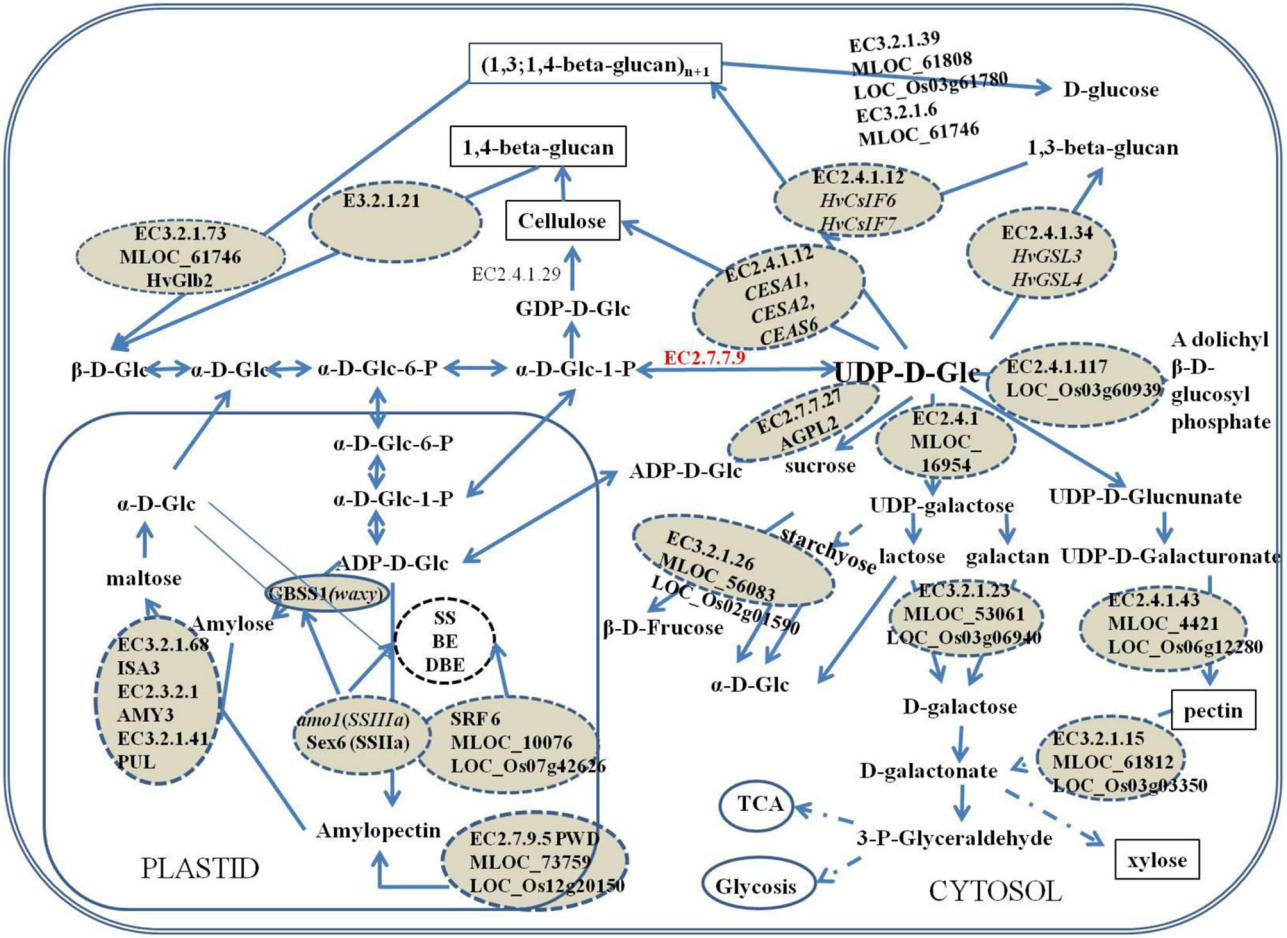
**Putative functions of the candidate genes in the co-regulated metabolite network of starch and β-glucan**. The candidate genes and their encoding protein were dashed circled. The cell wall components were marked by black box. The dash arrows indicated there were no a single step. EC3.2.1.73: (1,3; 1,4)-β-glucanase; EC3.2.1.21: β-1,4-glucosidase; EC3.2.1.39: glucan-1,3-β-glucosidase; EC3.2.1.6: glucan-1,3-β-glucosidase; EC2.1.4.12: Cellulose synthase; EC2.4.1.34: glucan synthase; EC2.4.1.117: dilichyl-phosphate-β-glycosyltransferase; EC2.4.1: galactosyl transferase; EC2.7.7.9: UTP–glucose-1-phosphate uridylyltransferase; EC2.7.7.27: ADP-glucose pyrophosphorylase; EC3.2.1.23: β-galactosidase; EC23.2.1.26: β-fructofuranosidase; EC2.4.1.43: glucosyl transferase; EC3.2.1.15: polygalacturonase; EC2.7.9.5: phosphoglucan water dikinase; EC3.2.1.68: Isoamylase; EC3.2.2.1: amylase; EC3.2.1.41: Amylopullulanase.

The significant SNPs associated with β-glucan content on chromosome 7H around 71 cM are within a 1–2 cM of the *HvCslF6* gene (Figure 3). *HvCslF6* was found essential for (1,3; 1,4)-β-D-glucan biosynthesis from UDP-glucose (Figure 4). Other significant SNPs for β-glucan content identified in this study are located on chromosome 4 and 6H. The gene containing SCRI_RS_156130 SNP on 4H is coding for glucan synthase-like 3 (EC 2.4.1.34, HvGSL3) which is known to be involved in the generation of β-glucan by catalyzing the transfer of sugar moieties from activated donor molecules to specific acceptor molecules to form glycosidic bonds. This gene showed 99% identity to *Brachypodium GSL3* gene (Bradi2g40460), and 83% identity to rice 1,3-β-glucan synthase gene (LOC_Os03g03610). *HvGSL3* belong to the callose synthase gene family of the GLS family and catalyzed the synthesis of 1,3-β-glucan (Figure 4). Another SNP (BOPA1_1565-514) at 66.08 cM on 6H shows synteny to a gene encoding 60S ribosomal protein L31 (MLOC_36568, LOC_Os06g21480 and Bradi3g53460) which catalyzes GTP to GDP hydrolysis in maize (www.gramene.org).

### Candidate genes for amylose content

Based on this study, and considering a LD of 4–6 cM (Tondelli et al., 2013), associated SNPs for amylose located on chromosome 1H are likely to be close to the *amo1* gene as they were all mapped within a 1–2 cM region (Figure 3). However, when analyzing the genes according to barley genome sequence, *amo1* gene is far from these SNPs, which can be due to mistakes in assembling of the particular barley contig or *amo1* is not the significant locus in this case. Within this genomic region, one (1,3;1,4)-β-glucanase gene (MLOC_61746) was found.

The SNP SCRI_RS_16118 located on chromosome 5H at 71 cM close to a glycogen debranching enzyme gene (MLOC_10776) which is almost identical to *HvISA3* and the other significant SNPs on chromosome 5H at the region of 152.36–155.42 cM was found syntenous with the region of rice genome containing several GT genes. In which LOC_Os03g60939, syntenous to SNP SCRI_RS_132308, codes for dolichyl-phosphate β-glycosyltransferase (EC2.4.1.117) belonging to the GT2 family which includes cellulose synthase acting on UDP-glucose and participating in n-glycan biosynthesis. Furthermore, *HvAGPL2* was found only 3458 bp upstream SCRI_RS_132308.

### Candidate genes for amylopectin content

For amylopectin content, the significant associated SNP SCRI_RS_98829 on chromosome 7H located at 15.37 cM is located in the starch synthase gene (MLOC_66457, GBSSI), also designated as *waxy*. It is essential for the synthesis of amylose and in addition it has a role in the elongation of amylopectin carbon chains (Denyer et al., 1996). The significant associations around 126 cM on chromosome 7 should be *Amy2* which catalyzes the hydrolysis of (1–4)-α-D-glucosidic linkages in starch degradation (Figure 3). *sex6* locus (*SSIIa*), one major gene responsible for amylopectin synthesis, was mapped around 126 cM on 7H according to barley genetic map OPA2005 (http://wheat.pw.usda.gov), however, according to its' physical position, *sex6* should be close to SCRI_RS_158266 and SCRI_RS_17908 located on 12.75 cM (Figure 3). These two SNPs are located in the phosphoglucan water dikinase gene (*PWD*, MLOC_73759). PWD catalyze the transfer of the β-phosphate of ATP to a glucosyl residue of amylopectin that has specifically been phosphorylated at the C-3 positions by α-glucan water dikinase (Kotting et al., 2009). The phosphorylation of a portion of the glucosyl residues is a prerequisite for transient starch mobilization. It still remains to show how PWD acts in cereal endosperm starch metabolism, whether the influence is directly on the amylopectin synthesis or indirectly by regulating amylopectin metabolism through interactions.

Another two SNPs SCRI_RS_208186 and BOPA1_7172-1536 at 1.49 cM on chromosome 7H were orthologous to rice genes encoding β-fructofuranosidase (EC 3.2.1.26) (LOC_Os02g01590) and polygalacturonase (EC3.2.1.15) (LOC_Os03g03350), respectively. Both enzymes belongs to the glycoside hydrolase family and β-fructofuranosidase catalyzes sucrose (sucrose-6P) or stachyose degradation to actuate D-glucose (glucose-6P) to recycle (Figure 4). LOC_Os07g42626 (SRF6, Leucine-rich repeat transmembrane protein kinase 1) ortholog to SNP SCRI_RS_6792 on chromosome 2H with the high *P*-value (Table 1) might also be very important for polysaccharide synthesis or transport. The SNP SCRI_RS_205669 on chromosome 1H was located on β-1,3-galactosyltransferase-like protein gene (MLOC_16945), orthologs to rice galactosyltransferase family protein gene (LOC_Os07g09670), which belong to glycosyl transferase family 31 and catalyzes the transfer of galactose.

### The interactions of candidate genes on biosynthesis of amylose, amylopectin and β-glucan

The SNP BOP2_12_30716 located on chromosome 5H detected for amylose codes for lysosomal alpha-mannosidase, 70 kb upstream from *HvCsIF7* which was confirmed to mediate the synthesis of cell wall components (Burton et al., 2006), furthermore, *CESA2* was downstream this gene (Figure 3). *HvCsIF7* and *HvCESA2* were confirmed to be responsible for the synthesis of cell wall 1,3;1,4-β-glucan and primary cell wall assembly which was the predominant cell wall components in endosperm cell (Burton et al., 2006; Held et al., 2008). Other genes like MLOC_61808 (BOPA1_6781-1073) encoding 1,3-β-glucosidase (EC3.2.1.39), hydrolyze 1,3-β-glucan to D-glucose (Figure 4) which is used in metabolism of starch, sucrose and other carbohydrates. Another candidate gene MLOC_61746 locating around 55.5 cM on chromosome 1H, also detected for amylose content, encoding (1,3;1,4)-β-glucanase (EC3.2.1.73), was functionally linked to the hydrolysis of (1,4)-β-D-glucan linkages and acts on lichenin and cereal β-D-glucans containing (1,3;1,4)-bonds (Figure 4).

Furthermore, the SNP at 2.55 cM on chromosome 7H detected for amylopectin was close to glucan synthase-like 8 gene which was very similar to *HvGSL4* and was mapped close to marker ABG704 at the intervals of 0–5 cM on the short arm of chromosome 7H (Schober et al., 2009). On its' syntenous rice genome region, the ortholog was also adjacent to one of the rice callose synthase genes (LOC_Os06g02260). The gene containing SNP BOPA1_3164_1386 on chromosome 6H codes for cellulose synthase-1, which shows 88.03% identity to rice *CESA1* gene (LOC_Os05g08370) and 89% identity to *HvCESA6*. *HvCESA6* shows potential functions in synthesis of cell wall cellulose and (1,3;1,4)-β-D-glucan (Held et al., 2008).

## Discussions

### Colorimetric method and correlations between amylose and β-glucan

Amylopectin was measured in parallel with amylose using a dual-wavelength colorimetric method, although the widely used method for quantifying amylopectin is by calculating the difference between total starch and amylose measured. The colorimetric method is easy to operated and economic in use and using dual wavelength has already been proven to be a valid method to measure amylopectin content (Wang et al., 2010). Though the amylose content determined with the I_2_/KI method showed a little higher value compared with those obtained by Megazyme Kit when analyzed several barley samples, there were positive correlations between the colorimetric method and Megazyme Kit (data not shown) and for genetic analysis was considered valid. The negative correlations between amylose and β-glucan is consistence with two previous studies based on a much small number of specialty barley lines. β-glucan was found to be negatively correlated with amylose in a study of 27 barley lines (Hang et al., 2007) as described in another study of 5 hull-less barley (Lee et al., 2011), while a study of ten hull-less barley lines showed that in addition to waxy type also high-amylose barley had higher β-glucan content compared to barley with normal amylose content (Asare et al., 2011).

### SNPs association with β-glucan, amylose and amylopectin

Barley, being self-pollinator, has a relative high LD of 4–6 cM based on a threshold *r*^2^ value of 0.15 making gene discovery by GWAS feasible (Comadran et al., 2012). Significant SNPs for β-glucan were distributed across chromosome 1, 4, 5, 6, and 7H. A QTL for β-glucan located on the short arm near the telomere (13.6–21.5 cM) on chromosome 4H between MWG077-Ole1 was mapped based on both “Morex” and “Steptoe” genetic backgrounds (Gao et al., 2004). The major SNPs region on chromosome 7H detected by present GWAS contains *HvCslF6*, which is consistent with other studies. One QTL mapped in a recombinant inbred line mapping population was also located in the region containing *HvCslF6* (Islamovic et al., 2012). A major QTL explaining up to 39% of the β-glucan concentration was reported to be located on the centromeric region of chromosome 7H (Li et al., 2008). Three associated SNP markers on chromosome 7H around 111 cM were identified with GWAS, among which *nud* (naked caryopses gene) locus should be the determiner for β-glucan content (Mezaka et al., 2011). The *nud* gene was found to be linked with the *bgl* gene which has been mapped to the centromeric region of chromosome 7H and the *bgl* (β-glucanless) phenotypes co-segregated with *HvCslF6* and caused by the mutation of *HvCslF6* (Tonooka et al., 2009).

For amylose content, a number of QTLs for starch or amylose content have also been mapped on chromosome 1, 5, and 7H in the same regions (Islamovic et al., 2012; Pasam et al., 2012) as the chromosome regions detected here.

Waxy locus associating to amylopectin in the present GWAS was earlier mapped between markers ABG320 and ABC151A located around 10.1–20.5 cM (http://wheat.pw.usda.gov) and produced a high level of amylopectin. Islamovic et al. (2012) also found one of three QTLs were mapped to chromosome 7H might be the *waxy* locus, using 297 markers in a population of 146 F_6_-derived RILs. Besides these major loci mapped before, two new SNPs located at 1.49 cM on chromosome 7H were first detected here. That indicate GWAS is effective in identifying major loci as well as detecting some new loci. Furthermore, some associated SNPs were overlapped among amylose, amylopectin and β-glucan, which indicated there should be some interactions among amylose, amylopectin and β-glucan metabolites.

### Candidate genes and their interactions

#### β-glucan

*HvCslF6* located around 71 cM on chromosome 7H should be the major gene responsible for β-glucan. Similarly, in a GWAS of oat one significant DArT marker sequence associated with β-glucan content was located in rice and found adjacent to an OsCslF gene (Newell et al., 2012). *HvCslF6* showed the highest transcript level throughout the endosperm development out of 7 *HvCslF* genes identified and characterized (Burton et al., 2006, 2008) and it was the key determinant for the biosynthesis of (1,3; 1,4)-β-D-glucan (Taketa et al., 2012). Over-expressing *HvCslF6* oat lines showed 80% higher (1,3; 1,4)-β-D-glucan content (Burton et al., 2011) and genetic markers for this gene had been applied successfully in segregating individuals with different β-glucan content (Cory et al., 2012).

Besides *HvCslF6, HvGSL3* located on chromosome 4H, were also detected in this study. The minor genes controlling β-glucan content seems to cluster together. As in the same syntenous region of *Brachypodium*, a cluster of genes involved in β-glucan synthesis including β-galactosidase (Bradi2g40450) and glucan synthase-like 11 (Bradi2g40437) can be found. Furthermore, downstream of *HvGSL3*, a glucan synthase-like gene *HvGSL1* has been mapped on 107.7 cM (Schober et al., 2009), corresponding to about 130 cM in the SNP map of OPA 2009 (http://wheat.pw.usda.gov).

#### Amylopectin

In the present GWAS, we obtained several genes responsible for amylopectin content including *waxy*. The *waxy* gene is epistatic over the *amo1* gene and its mutation affected the activity of GBSSI greatly (Hylton et al., 1996), which means that the loss or the reduced activity of GBSS1 can result in a waxy phenotype with high amylopectin content, while its mutation did not abolish amylose synthesis completely in the starchy endosperm but reduced the amylose content below 5% (Andersson et al., 1999).

PWD, one of the new genes identified for amylopectin in this study, exclusively phosphorylates the C-3 positions of starch (Kotting et al., 2009). Phosphorylation of starch disrupts the packing of amylopectin double helices and can render them accessible for the subsequent degradation by enzymes like isoamylase (ISA), pullanase (PUL), and alpha-/beta- amylase (AMY, BMY) (Zeeman et al., 2010) (Figure 4) and influences amylopectin accumulation. Reduced PWD expression in transgenic Arabidopsis can result in excess starch phenotype (Kotting, 2005; Kotting et al., 2009).

β-fructofuranosidase might play an important role in starch synthesis especially in the amylopectin pathway because its orthologous SNP SCRI_RS_208186 had the highest *P*-value for amylopectin content (Table 1). Though the function of SRF6 remained elusive, it might act as the key protein kinase to catalyze the phosphorylation of those enzymes, like SS and SBE, participating in carbohydrate metabolism (Figure 4). It was found that enzymes for amylopectin synthesis were regulated by protein phosphorylation which played a wide role in controlling starch metabolism in wheat amyloplasts (Tetlow et al., 2004) and starch synthesis is regulated through the interactions of a number of genes and regulators, thus different combinations of mutants resulted in different amylose content and amylose to amylopectin ratio. As an example a double mutant *amo1sex6* showed parallel increases in both amylose and amylopectin content (Li et al., 2011).

### Interactions among amylose, amylopectin and β-glucan

In this study, several genes belonging to GT family were identified to be associated with amylose and amylopectin content. Members of the GT family and glycoside hydrolase were also found to be responsible for resistant starch accumulation in a recent GWAS of barley (Shu et al., 2012). That indicated these genes might affect synthesis of starch molecular components indirectly by regulating other pathways in the metabolic network through the same substrates especially those depending on UDP-glucose (Figure 4). In cereals like barley, other major carbohydrates of endosperm are cell wall polysaccharides of which β-glucan is the primary component.

Chromosomes 5 and 7H contain major QTLs and genes controlling amylose, amylopectin and β-glucan in barley. The overlapping associations among these three traits might suggest that polysaccharides like starch and β-glucan in barley endosperm are correlated due to shared genetics and may have negative or positive pleiotropic effects. UDP-glucose is a central substrate in these metabolic pathways, the mutation of one single gene or in one single step may cause dramatic changes in the whole metabolic network a new area for pleiotropic mechanism (Stearns, 2010). Several minor genes might affect synthesis of starch fractions by regulating the formation of the cell wall polysaccharides constituents, while how they work and interact needs further studies. Chromosome 7H, on which several genes responsible for starch and β-glucan were closely located as an operon like structure, seems to be an important chromosome for barley grain quality. These results showed that, with GWAS, major genes or genetic regions as well as two new genes important for quality traits in cereal crops can be identified successfully. This approach can also help to dissect element in interaction network resulting in different traits, interactions that often has been said to be pleiotropic effects. The highly associated SNPs distributed one several chromosomes especially those within the candidate genes are a first choice for testing the power of genomic selection in barley.

### Conflict of interest statement

The authors declare that the research was conducted in the absence of any commercial or financial relationships that could be construed as a potential conflict of interest.

## Supplementary material

Table S1 **The AAC, amylopectin and beta-glucan content in all analyzed 254 European spring barley varieties**.

## Abbreviations

AGPase: ADP-Glc pyrophosphorylase
CESA: Cellulose synthase A
CSL: cellulose synthase-like
GBSSI: granule-binding starch synthase I
GDP: guanosine diphosphate
GSL: glucan synthase-like
GT: glycosyltransferase
GTP: guanosine triphosphate
GWAS: genome wide associatioin scan
LD: linkage disequilibrium
QTL: quantitative trait loci
SBE: starch branching enzyme
SNP: single nucleotide polymorphism
SS: starch synthase
UDP: uridine diphosphate.
