# Supplementary material for: Quantification of amylose, amylopectin, and β-glucan in search for genes controlling the three major quality traits in barley by genome-wide association studies
Source: Front Plant Sci. 2014 May 15;5:197. doi: 10.3389/fpls.2014.00197 (PMC4030205; doi:10.3389/fpls.2014.00197)
Supplement: Table S1 — The AAC, amylopectin and beta-glucan content in all analyzed 254 European spring barley varieties. [file Table1.PDF]

Table S1. The AAC, amylopectin and beta-glucan in all analyzed 254 European spring barley germplasm

Cultivar	Beta-glucan (%)	AAC (%)	Amylopectin (%)	Type
**Ingrid**	2.08±0.077	23.41±3.09	35.71±5.59	2
**POHTO**	1.75±0.047	25.76±0.22	40.32±1.87	2
**Hanka**	1.75±0.07	26.16±0.79	40.32±2.81	2
**FORMULA**	2.57±0.02	26.24±2.47	39.66±3.75	2
**Optic**	1.63±0.04	29.02±1.01	42.97±2.81	2
**Prisma -B**	2.09±0.09	26.39±0.0	42.97±0.00	2
**Ametyst**	1.89±0.05	22.98±1.24	39.33±1.41	2
**Atem**	2.37±0.01	24.88±0.34	39.33±0.47	2
**Tyra - A**	2.73±0.07	22.98±0.11	35.35±0.47	2
**Gerkra**	2.16±0.05	24.80±0.67	37.34±0.47	2
**Salve**	2.76±0.04	20.12±1.46	33.70±2.81	2
**Binder**	2.40±0.07	21.78±0.45	34.36±0.94	2
**Gull**	2.23±0.02	20.59±0.34	32.37±0.94	2
**Riviera**	2.62±0.01	20.91±0.79	30.71±1.41	2
**Class**	2.06±0.04	25.36±0.11	37.34±0.47	2
**Braemar**	1.66±0.01	28.14±0.22	39.66±2.81	2
**Birgitta-A**	2.84±0.11	23.77±0.34	31.71±0.94	2
**Famin**	1.49±0.12	25.68±0.34	36.35±0.94	2
**Priekulu 60**	2.62±0.07	21.76±0.43	30.30±2.22	2
**Digersano (Naked)**	2.08±0.04	22.15±0.11	31.55±0.44	2
**Calgary**	2.38±0.10	22.83±0.43	30.30±0.44	2
**Leeni**	2.73±0.00	21.63±0.67	32.37±0.00	2
**Gitane**	2.16±0.15	24.96±0.00	34.36±0.94	2
**Akcent**	1.91±0.04	25.44±1.35	37.34±2.34	2
**Tremois**	2.38±0.06	24.49±0.45	34.03±2.34	2
**Frisia**	2.35±0.04	23.35±0.10	27.79±2.22	2
**Annabell**	1.73±0.09	24.01±2.7	30.39±0.94	2
**Isaria**	2.56±0.08	22.42±0.45	31.71±0.00	2
**Elo**	2.46±0.05	22.26±2.47	31.38±3.28	2
**Terno**	2.09±0.03	21.23±0.34	31.05±0.00	2
**PALLAS**	2.44±0.11	26.16±1.01	36.68±2.34	2
**Olli**	2.76±0.06	22.26±0.22	31.38±0.47	2
**Jarek**	2.63±0.12	21.76±0.43	31.23±0.89	2
**Lofa Abed**	2.17±0.09	22.42±3.82	31.05±5.62	2
**Zenit**	2.37±0.12	21.94±0.22	30.39±1.87	2
**Hanna (W273368)**	2.54±0.08	20.39±0.65	31.55±1.33	2
**Barke**	2.05±.05	26.31±1.01	35.02±1.87	2
**Golf**	2.59±0.02	22.26±0.22	31.38±0.47	2
**AKKA (W 6039)**	1.88±0.09	25.52±0.33	32.87±1.17	2
**Agra**	2.21±0.05	24.17±1.12	33.04±0.94	2
**Cristalia**	2.45±0.16	25.50±0.54	29.98±0.89	2

**Priora (Naked)**	2.58±0.02	23.14±0.11	31.05±0.00	2
**Niina**	1.84±0.02	29.84±1.29	36.56±1.33	2
**Gizmo**	2.14±0.13	24.13±0.97	31.23±0.89	2
**Camir**	2.71±0.09	25.34±0.54	35.31±0.44	2
**Dialog**	2.19±0.15	25.04±0.32	31.86±0.89	2
**SILJA**	2.15±0.09	25.95±0.32	31.55±1.33	2
**POMO**	2.21±.07	25.72±1.29	33.11±0.89	2
**ASPLUND-A**	2.09±0.04	26.11±0.75	33.11±0.89	2
**KARRI**	2.24±0.07	26.64±0.00	34.05±0.44	2
**Alliot**	2.17±0.16	27.17±0.11	33.11±0.89	2
**Primus**	1.85±0.10	26.56±0.75	35.62±1.77	2
**Otra**	2.42±0.07	23.21±0.75	31.23±1.77	2
**vankkuri**	1.99±0.07	28.47±1.08	34.37±0.89	2
**Scandium**	1.77±0.07	26.49±0.43	33.74±0.89	2
**Steffi**	1.52±0.07	26.79±1.29	34.05±1.33	2
**Prosa**	2.02±0.11	27.17±1.18	30.61±0.89	2
**BALDER**	2.30±0.01	23.36±0.32	30.92±0.44	2
**Rapid**	2.12±0.04	20.85±0.43	31.23±0.89	2
**ARTTUR(Artivri)**	2.10±0.09	25.19±0.11	32.49±0.89	2
**NFC Tipple**	2.39±0.11	25.50±0.54	33.43±0.44	2
**Abacus**	2.38±0.05	24.81±0.43	32.17±0.44	2
**Spartan**	2.65±0.05	20.01±0.11	29.98±0.00	2
**Aapo**	3.2±0.12	16.74±0.00	24.66±1.33	2
**Elantra**	2.72±0.08	21.46±1.94	33.11±2.66	2
**Esme**	2.01±0.06	26.64±0.86	33.43±1.33	2
**Cleopatra**	2.54±0.02	20.85±0.65	26.54±2.22	2
**Power**	1.63±0.10	28.62±0.43	35.31±2.22	2
**Otto**	2.59±0.03	25.42±0.86	33.11±0.89	2
**Cabaret**	2.34±0.06	28.31±0.65	37.81±2.22	2
**Odessa - B**	2.09±0.06	24.80±0.00	34.69±0.47	2
**Golden Promise**	2.26±0.04	24.89±0.75	33.11±1.77	2
**Dina**	2.58±0.04	20.43±1.69	28.40±1.87	2
**Wisa**	2.35±0.02	23.53±1.57	33.70±3.75	2
**Alexis**	2.37	24.58±0.11	30.61±0.89	2
**Simon**	2.56±0.10	20.91±1.91	29.39±4.22	2
**Carlsberg**	2.65±0.11	21.94±2.02	31.05±3.75	2
**Delta**	2.88±0.07	23.93±2.36	33.37±2.34	2
**Eunova**	2.55±0.05	25.76±0.22	33.37±3.28	2
**Rubin**	2.58±0.02	22.66±1.24	31.05±0.94	2
**Clara (W5690)**	2.23±0.07	22.74±0.00	30.39±0.94	2
**BIRKA**	2.69±0.02	24.05±1.07	32.72±0.55	2
**Nemex**	2.59±0.06	22.30±0.11	32.17±0.44	2
**Cooper**	1.45±0.03	27.67±0.22	40.32±0.00	2
**Tarm92**	2.61±0.05	21.31±0.45	33.70±0.94	2

**Isabella**	2.15±0.07	27.10±1.08	31.55±2.22	2
**Corgi**	2.55±0.06	24.73±0.75	31.86±2.66	2
**Abava**	2.19±0.09	25.68±0.79	36.68±0.47	2
**WELAM**	2.71±0.09	19.81±1.02	26.22±6.2	2
**Imula**	2.68±0.02	21.15±0.45	30.72±0.47	2
**Kenia**	2.58±0.14	22.53±1.08	32.49±0.00	2
**RIKA**	1.84±0.08	26.47±1.24	38.34±0.00	2
**Tidone**	2.53±0.02	23.61±1.01	33.37±1.41	2
**Rasa**	2.51±0.02	21.07±2.36	31.38±1.41	2
**Gant**	2.57±0.09	21.47±0.22	31.71±0.00	2
**Simba**	2.02±0.04	27.40±0.43	36.25±0.00	2
**Dandy**	2.30±.05	26.79±1.72	32.17±0.44	2
**Chanell**	2.04±0.10	26.56±0.75	32.49±0.89	2
**Sundance**	2.67±0.08	25.72±0.65	33.74±0.89	2
**Salka**	2.61±0.06	22.98±0.22	28.73±0.00	2
**Cameo**	2.83±0.09	23.52±1.62	31.23±2.66	2
**Meltan**	2.13±0.04	24.81±0.43	32.49±0.89	2
**Apex**	2.82±0.12	24.43±1.4	33.43±1.33	2
**Hana**	2.47±0.12	24.13±1.4	35.62±0.89	2
**Forum**	2.41±0.07	24.20±0.65	32.49±0.89	2
**Chalice**	2.08±0.11	25.95±2.48	33.43±3.1	2
**Nordal**	2.34±0.05	24.73±0.97	30.92±0.44	2
**Okos**	2.24±0.01	23.90±1.08	31.23±0.00	2
**Imber**	2.39±0.09	20.51±2.92	29.39±4.22	2
**Athos**	2.26±0.05	23.77±1.01	36.02±0.47	2
**VALTICKY**	2.25±0.08	25.44±1.35	37.01±0.94	2
**Caminant**	2.21±0.09	23.29±0.43	29.67±1.33	2
**Tocada**	2.17±0.09	25.15±0.2	30.30±1.33	2
**Nathalie**	1.82±0.01	24.73±1.01	36.68±2.34	2
**CILLA**	2.07±0.13	26.41±0.32	32.49±0.89	2
**Orbit**	2.66±0.05	22.82±0.34	33.04±1.87	2
**Diamant**	2.01±0.01	27.48±1.18	34.37±1.77	2
**Favorit**	2.30±0.04	24.84±0.17	33.02±2.53	2
**Mauritia**	1.74±0.03	29.15±1.4	35.62±0.89	2
**Quench**	1.76±0	29.84±0.22	34.05±0.44	2
**Galan**	1.93±0.01	26.37±0.7	32.77±3.94	2
**Static**	2.54±0.06	27.40±0.00	31.55±0.44	2
**Auriga**	1.95±0.07	27.17±0.97	33.11±0.89	2
**Triumph**	2.36±0.07	25.68±1.24	37.34±4.22	2
**Linga**	2.62±0.11	25.04±3.26	40.99±0.00	2
**Sultan**	2.56±0.05	20.32±0.32	29.98±0.89	2
**Felicitas**	1.69±0.04	28.14±2.7	42.64±6.09	2
**Carvilla**	2.16±0.10	23.61±1.01	33.70±1.87	2
**Proctor**	2.22±0.06	23.61±2.36	34.36±2.81	2

**Ladik**	1.96±0.05	27.27±2.14	36.39±0.94	2
**Quartz**	2.08±0.10	25.60±0.9	35.02±1.87	2
**Lysimax**	1.59±0.04	23.06±0.67	32.04±0.47	2
**Maja**	2.09±0.01	23.10±0.39	32.04±0.47	2
**Barabas**	2.65±0.07	24.09±0.34	32.37±0.94	2
**Ansis**	2.52±0.03	23.85±0.45	31.71±1.87	2
**MONA**	2.49±0.03	23.61±2.81	33.70±5.62	2
**Latvijas Vietejie**	2.89±0.05	21.07±0.11	35.69±0.00	2
**Drake**	2.80±0.05	21.46±0.23	35.36±0.47	2
**Scarlett**	2.40±0.11	21.55±1.01	34.69±3.28	2
**Hassan**	2.79±0.10	22.22±0.22	28.42±0.44	2
**Baronesse**	2.07±0.08	26.47±0.56	40.99±0.00	2
**Lenta**	2.83±0.07	23.37±1.12	35.02±3.75	2
**Steina**	1.97±0.07	24.66±0.65	31.86±1.77	2
**Atribut**	1.92±0.05	24.96±0.22	30.61±0.89	2
**Ilga**	2.64±0.13	23.59±2.15	33.11±3.54	2
**Doublet**	1.76±0.03	24.89±1.18	30.30±2.22	2
**Britta A**	2.58±0.04	23.14±0.86	31.86±0.89	2
**Volla**	1.92±0.03	24.91±1.37	33.74±0.88	2
**ALVA**	2.1±0.00	22.45±0.32	30.61±0.00	2
**Drost**	2.21±0.01	24.66±0.22	32.17±0.44	2
**Cheri**	1.59±0.05	20.62±0.32	30.92±0.44	2
**Imidis**	1.59±0.04	27.55±0.22	32.17±0.44	2
**Corniche**	1.60±0.04	27.25±0.43	34.05±2.22	2
**ARVO**	1.87±0.07	23.14±0.22	30.30±0.44	2
**Koral**	2.11±0.05	24.01±0.22	36.02±1.41	2
**Beatrix**	1.59±0.03	28.14±1.35	36.35±3.75	2
**Maris Mink**	2.18±0.15	23.74±0.22	32.80±0.44	2
**DOMEN**	2.20±0.06	24.73±0.54	30.61±0.89	2
**HELLAS**	2.72±0.04	22.37±2.58	27.16±3.99	2
**ROLAND**	2.25±0.12	23.61±1.05	32.04±1.41	2
**Otis**	1.95±0.33	20.01±0.54	27.16±0.44	2
**Malva**	2.02±0.02	22.60±0.75	29.36±0.89	2
**Aramir**	2.09±0.00	23.69±1.35	32.37±2.81	2
**Roxana**	1.63±0.04	21.15±0.9	33.37±0.47	2
**Emir**	2.41±0.05	23.74±0.65	28.73±0.89	2
**Heris**	2.26±0.08	23.93±1.69	36.02±0.47	2
**Union**	2.35±0.01	25.04±1.18	31.86±1.77	2
**Mars**	2.19±0.03	26.18±0.86	34.37±0.89	2
**Smilla**	2.18±0.04	27.25±0.22	35.31±0.44	2
**Egmont**	2.29±0.03	25.11±0.22	31.86±0.89	2
**Anni**	2.51±0.03	26.11±0.11	33.43±0.44	2
**Chariot**	1.34±0.01	30.05±0.22	40.72±0.00	2
**Midas**	2.02±0.02	26.26±0.54	31.55±1.33	2

**VIIVI**	2.98±0.06	21.69±0.11	28.10±0.89	2
**Bulbul 89**	2.92±0.08	22.15±0.97	32.49±0.00	2
**Zephyr**	2.82±0.14	19.18±1.72	28.10±1.77	2
**Kristaps**	2.17±0.01	23.21±0.11	32.49±0.89	2
**Safir**	2.57±0.01	23.90±0.86	32.80±2.22	2
**INARI**	2.10±0.08	27.19±0.9	39.66±2.81	2
**Karat**	1.47±0.04	29.65±0.79	38.90±0.94	2
**Mentor**	1.63±0.06	28.62±0.9	39.66±0.94	2
**Carafe**	1.42±0.06	29.97±0.56	40.66±0.47	2
**Brazil**	1.47±0.12	29.02±0.79	41.65±2.81	2
**Harry**	1.81±0.00	27.03±1.8	38.67±1.41	2
**Berenice**	2.29±0.04	24.25±2.14	35.36±4.22	2
**Bomi**	2.22±0.07	22.42±0.00	34.69±0.47	2
**Lux**	1.58±0.02	26.79±0.11	40.32±0.00	2
**Armelle**	1.84±0.11	26.16±0.56	38.34±0.94	2
**Marthe**	1.62±0.05	26.16±0.79	35.69±1.87	2
**Gorm**	2.03±0.07	25.52±1.01	35.02±0.00	2
**Derkado**	1.83±0.04	26.95±1.01	38.90±1.87	2
**URSEL**	1.41±0.02	26.71±0.9	38.34±0.94	2
**TELLUS (W 5897)**	2.17±0.07	23.53±0.00	36.02±0.47	2
**Odin**	1.94±0.03	26.47±0.56	39.33±0.47	2
**Mala**	1.98±0.07	26.08±0.45	39.00±1.87	2
**Vada**	2.02±0.08	25.84±0.56	39.33±0.47	2
**Atlas**	1.60±0.08	24.73±1.01	38.01±1.41	2
**Alis**	1.74±0.10	25.84±1.46	39.0±1.87	2
**Ida**	1.85±0.09	25.28±0.45	37.67±0.00	2
**Ceylon**	1.74±0.06	25.52±1.01	37.34±2.34	2
**Ruja**	1.61±0.08	28.38±0.56	37.34±1.41	2
**Perun**	1.87±0.07	25.20±0.34	38.34±1.87	2
**Paloma**	2.36±0.05	23.29±0.34	34.03±1.41	2
**Hydrogen**	1.52±0.06	26.16±0.11	36.68±1.41	2
**Chamant**	1.81±0.04	28.24±0.54	36.87±0.00	2
**Cicero**	1.83±0.00	26.18±0.86	32.80±3.10	2
**Idumeja**	1.97±0.01	26.08±1.12	33.62±0.91	2
**Gate**	2.31±0.03	23.59±0.65	33.43±1.33	2
**Claude**	1.97±0.07	24.72±0.56	33.74±2.66	2
**SAANA**	2.36±0.12	23.74±0.65	30.61±0.00	2
**Mari**	1.89±0.06	27.40±0.00	35.31±1.33	2
**Pasadena**	2.15±0.06	24.01±1.12	33.03±1.89	2
**GUNILLA**	2.07±0.09	24.57±1.91	38.68±4.22	6
**VEGA**	2.35±0.03	24.05±1.29	31.55±1.33	6
**Krona**	2.30±0.09	23.52±1.18	30.61±1.77	6
**LISE**	2.24±0.13	24.89±1.18	31.23±0.00	6
**EDDA**	1.57±0.02	27.10±1.08	33.43±3.1	6

**Local 2**	2.58±0.07	23.02±0.05	30.83±1.21	6
**VARDE**	2.23±0.07	21.63±01.35	31.05±3.75	6
**Magda**	2.36±0.01	23.14±1.01	30.72±1.41	6
**ARRA**	1.94±0..11	26.71±0.67	35.36±0.47	6
**Rondo (Naked)**	2.94±0.07	19.16±0.11	32.37±0.94	6
**Steptoe**	2.85±0.02	22.37±0.43	30.30±0.44	6
**KILTA**	1.98±0.39	27.10±0.22	34.37±0.89	6
**PIRKKA**	1.70±.04	22.75±0.54	27.79±1.33	6
**HERSE**	2.19±0.09	21.94±1.80	33.04±2.81	6
**TAMMI**	2.26±0.02	24.57±3.03	35.69±2.81	6
**Morex**	2.16±0.01	23.93±0.56	35.02±0.94	6
**Anla**	2.54±0.15	25.50±1.18	34.37±0.89	6
**ROLFI**	2.18±0.06	25.28±0.00	38.01±0.47	6
**TEEMU**	2.78±0.06	23.14±0.65	31.86±0.00	6
**Priekulu 1**	2.14±0.01	24.41±0.56	35.35±0.47	6
**Pokko**	2.02±0.03	23.52±0.32	30.30±0.44	6
**Sladar**	2.37±0.07	23.59±0.00	29.98±0.00	6
**JADAR**	1.64±0.12	24.49±0.45	32.37±0.00	6
**Klinta**	1.90±0.02	23.36	28.73±0.00	6
**Local 1**	2.70±0.10	23.97±0.32	34.05±0.44	6
**Vairogs**	2.04±0.13	19.94±0.65	27.48±1.77	6
**STELLA**	1.91±0.05	23.37±0.00	35.67±0.00	6
**Local 3**	2.20±0.03	27.63±0.11	34.37±0.00	6
**Impala**	2.06±0.06	26.79±0.22	34.05±0.44	6
**ERKKI**	2.47±0.05	24.81±1.08	30.92±1.33	6
**Zita**	2.73±0.02	21.76±0.65	29.36±0.00	6
**Ark Royal**	2.49±0.11	21.07±1.01	31.71±1.87	6
**Diomede**	1.53±0.11	25.36±0.11	37.67±0.94	6
**agneta**	1.49±0.09	30.92±0.11	38.90±0.00	6
**MASKIN**	1.80±0.07	23.77±1.01	36.02±2.34	6
**PAAVO**	1.90±0.09	24.96±0.45	37.01±0.94	6
**BOTNIA**	1.66±0.04	22.79±0.15	32.17±2.22	6
**SUVI**	2.25±0.04	23.52±0.75	29.36±0.89	6
**KAJSA**	1.73±0.09	24.20±1.29	30.92±1.33	6
**Druvis**	2.30±0.05	26.79±0.22	34.68±0.44	6
**LOVIISA**	2.22±0.02	24.89±1.18	31.23±2.66	6
**Jyvä**	1.83±0.04	26.87±0.11	34.05±0.44	6
